# Pancreatic β-cell hyper-O-GlcNAcylation leads to impaired glucose homeostasis *in vivo*

**DOI:** 10.3389/fendo.2022.1040014

**Published:** 2022-10-26

**Authors:** Seokwon Jo, Samantha Pritchard, Alicia Wong, Nandini Avula, Ahmad Essawy, John Hanover, Emilyn U. Alejandro

**Affiliations:** ^1^ Department of Integrative Biology & Physiology, University of Minnesota Medical School, Minneapolis, MN, United States; ^2^ Department of Genetics, Cell Biology & Development, University of Minnesota, Minneapolis, MN, United States; ^3^ Laboratory of Cell and Molecular Biology, National Institute of Diabetes and Digestive and Kidney Diseases (NIDDK), National Institutes of Health, Bethesda, MD, United States

**Keywords:** O‐linked N‐acetylglucosamine (O‐GlcNAc), O-GlcNAcylation, O-GlcNAcase (OGA), beta cell (β‐cell), insulin, insulin secretion, high fat diet (HFD), Streptozocin (STZ)

## Abstract

Protein O-GlcNAcylation is a nutrient and stress-sensitive protein post-translational modification (PTM). The addition of an O-GlcNAc molecule to proteins is catalyzed by O-GlcNAc transferase (OGT), whereas O-GlcNAcase (OGA) enzyme is responsible for removal of this PTM. Previous work showed that OGT is highly expressed in the pancreas, and we demonstrated that hypo-O-GlcNAcylation in β-cells cause severe diabetes in mice. These studies show a direct link between nutrient-sensitive OGT and β-cell health and function. In the current study, we hypothesized that hyper-O-GlcNAcylation may confer protection from β-cell failure in high-fat diet (HFD)-induced obesity. To test this hypothesis, we generated a mouse model with constitutive β-cell OGA ablation (βOGAKO) to specifically increase O-GlcNAcylation in β-cells. Under normal chow diet, young male and female βOGAKO mice exhibited normal glucose tolerance but developed glucose intolerance with aging, relative to littermate controls. No alteration in β-cell mass was observed between βOGAKO and littermate controls. Total insulin content was reduced despite an increase in pro-insulin to insulin ratio in βOGAKO islets. βOGAKO mice showed deficit in insulin secretion *in vivo and in vitro.* When young animals were subjected to HFD, both male and female βOGAKO mice displayed normal body weight gain and insulin tolerance but developed glucose intolerance that worsened with longer exposure to HFD. Comparable β-cell mass was found between βOGAKO and littermate controls. Taken together, these data demonstrate that the loss of OGA in β-cells reduces β-cell function, thereby perturbing glucose homeostasis. The findings reinforce the rheostat model of intracellular O-GlcNAcylation where too much (OGA loss) or too little (OGT loss) O-GlcNAcylation are both detrimental to the β-cell.

## Introduction

Protein O-GlcNAcylation is a nutrient and stress-sensitive protein post-translational modification (PTM). The addition of an O-GlcNAc molecule onto proteins is catalyzed by O-GlcNAc transferase (OGT), whereas O-GlcNAcase (OGA) enzymes are responsible for removal of this PTM. O-GlcNAcylation occurs in intracellular proteins in the cytosol, mitochondria, and nucleus of the cell (1). The substrate, UDP-N-acetylglucosamine (UDP-GlcNAc) is synthesized from the hexosamine biosynthetic pathway that incorporates major components of macronutrients (glucose, amino acids, lipid and nucleotides), integrating the nutrient status of the cell into intracellular response to regulate key cellular functions (2). Dysregulation of O-GlcNAc cycling has been associated with various pathophysiologies (3, 4), in particular, metabolic disorders such as diabetes (5).

OGT is highly expressed in the pancreas (6) and has distinctive effects in the pancreas, compared to other insulin-sensitive metabolic tissues (7). O-GlcNAcylation has been detected in embryonic pancreas (8) and has shown to be critical to maintain both endocrine and exocrine health and function. OGT loss or hypo-O-GlcNAcylation in pancreatic progenitors causes pancreatic hypoplasia (8). OGT deletion in β-cells or in α-cells reduces cell survival and function (7, 9, 10), thereby dysregulating glucose homeostasis *in vivo*. Islet O-GlcNAcylation is increased in early obesity and is required for β-cell adaptation to enhance insulin secretion in early states of obesity (11). More importantly, we showed that OGT expression and activity are reduced in human islets from donors with chronic obesity, and lower OGT expression is associated with reduced β-cell function (11). These studies show a direct link between nutrient-sensitive OGT and β-cell health and function.

O-GlcNAcylation has been implicated in the regulation of β-cell health and function by fine-tuning signaling pathways relevant to cell survival such as the UPR ER stress and mitochondrial function (12). In β-cells, OGT loss increases ER (10) and mitochondrial stress (12). Several OGT protein targets in β-cells have been identified including the master regulator Pdx1 (13, 14) (8), and others such as p53 (8), NeuroD1 (15), TxNIP (16) and eIF4G1 (17). The cycling of O-GlcNAcylation on these target proteins is balanced by the enzyme OGA. SNP in OGA gene is a type 2 diabetes susceptibility gene in humans (18). Currently, there are no known studies that aim to study the O-GlcNAc cycling specifically in the pancreatic β-cell and its subsequent effect on glucose homeostasis *in vivo*.

The perturbations in *O*-GlcNAc cycling have been studied *in vivo* using whole body and conditional deletion of OGA (19, 20). These studies revealed that complete deletion of OGA led to perinatal lethality, whereas heterozygosity of OGA deletion led to viable mice with perturbed metabolic phenotypes. Whole body heterozygosity of OGA in male mice led to lower body weight with improved glucose tolerance and these mice resisted high fat diet (HFD) induced weight gain associated with increased energy expenditure through brown adipose enhancement. In a separate study of conditional deletion of OGA through MMTV-Cre recombinase, the authors reported sexual dimorphism phenotypes: Males displayed normal body weight, glucose tolerance (GTT) and insulin secretion *in vivo*, while female mice exhibited greater body weight gain (increase in both lean and fat mass), normal GTT, and increased insulin secretion *in vivo* (19). Under metabolic stress (HFD), OGA deletion led to improved GTT and normal insulin secretion in male mice, and glucose intolerance in females despite improved insulin secretion *in vivo*. In this study, however, insulin sensitivity was not tested.

Previous reports investigating the requirements of OGA provided important information about the metabolic effects of altered *O*-GlcNAc cycling in the whole animal (19). However, these studies are limited due to deletion of OGA in various tissues with possible conflicting effects in different tissues. To study the effect of blunted O-GlcNAc cycling in β-cell health and function and its impact on whole body glucose homeostasis, we characterized the mouse model of pancreatic β-cell specific OGA deletion. We reveal age-dependent impairment in glucose tolerance in both male and female in βOGAKO mice under normal chow diet, and that glucose intolerance in these mice was associated with defects in insulin content and insulin secretion. In response to a high-fat diet, male and female βOGAKO mice developed worse glucose intolerance. Under the diabetogenic stressor, streptozocin, βOGAKO mice developed hyperglycemia like littermate controls. The present study highlights the importance of O-GlcNAcylation cycling in both male and female animals and its implication in β-cell failure in diabetes.

## Results

### Generation of pancreatic β-cell specific OGA deletion in mice

**Figure 1:**
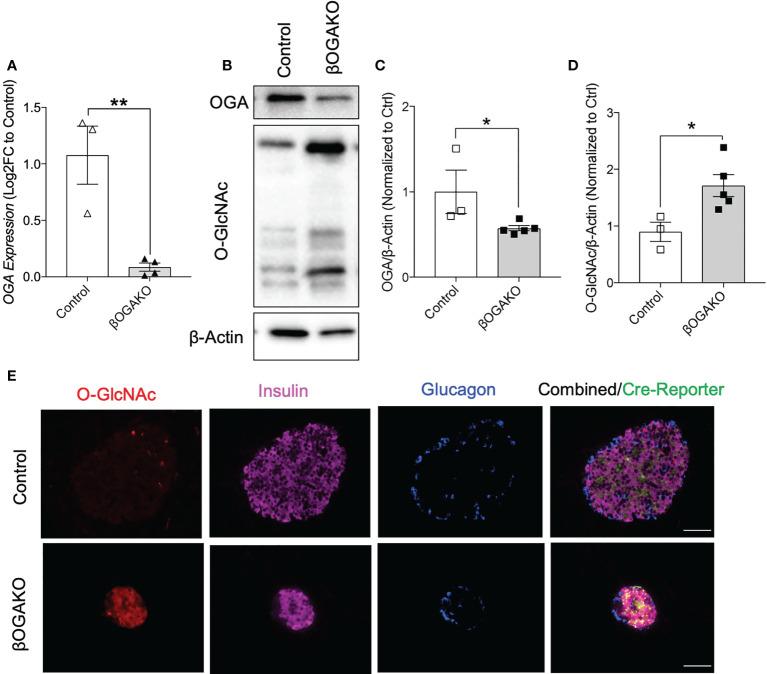
Validation of the β-Cell Specific Deletion of OGA. OGA mRNA level from pancreatic islets of control and βOGAKO mice **(A)**, normalized to beta-Actin mRNA (n=3-4). Representative western blot **(B)** and quantification of OGA **(C)** and pan O-GlcNAcylation **(D)**, normalized to beta-Actin (n=3-5). Immunofluorescence image of pancreatic islets showing O-GlcNAcylation (Red), Insulin (Purple), Glucagon (Blue) and Cre Reporter (Green) in Control and βOGAKO pancreas **(E)**. 40x magnification. Scale bar = 50 μm. Statistical analyses were conducted using unpaired, 2-way student t-test with significance *p<0.05, **p<0.001.

OGA, a key enzyme responsible for the removal of post-translational modification of O-GlcNAcylation, is a T2D susceptible gene in humans (21). To assess the role of OGA in the pancreatic β-cells and its subsequent contribution to glucose homeostasis, we generated a mouse model with β-cell specific ablation of OGA using the Rat-Insulin-Promoter driven Cre-recombinase and OGA floxed gene (RIP-cre; OGA flox/flox, herein referred to as βOGAKO). We confirmed that the isolated islets (containing β-cells and other endocrine cells) from βOGAKO mice show significantly lower OGA mRNA transcript and protein levels and increased protein O-GlcNAcylation levels than control islets (**Figures 1A-D**). Interestingly, we show reduced protein levels of OGT, without alterations to its transcript levels in βOGAKO islets (**Supplemental Figures 1A, B** **)**, suggesting that O-GlcNAc cycling enzyme expression is sensitive to overall O-GlcNAc status of the cell. Since pancreatic islets consist of other endocrine cell types that express OGA and the O-GlcNAc-modified proteins (e.g., α-cells), we validated the specificity of the deletion using immunofluorescence imaging. Here, we observed an increase in O-GlcNAcylation specifically in insulin producing β-cells (**Figure 1E**), validating the specificity of OGA deletion in βOGAKO mice.

### OGA deletion in pancreatic β-cell perturbs glucose homeostasis under standard chow in age-dependent manner

**Figure 2:**
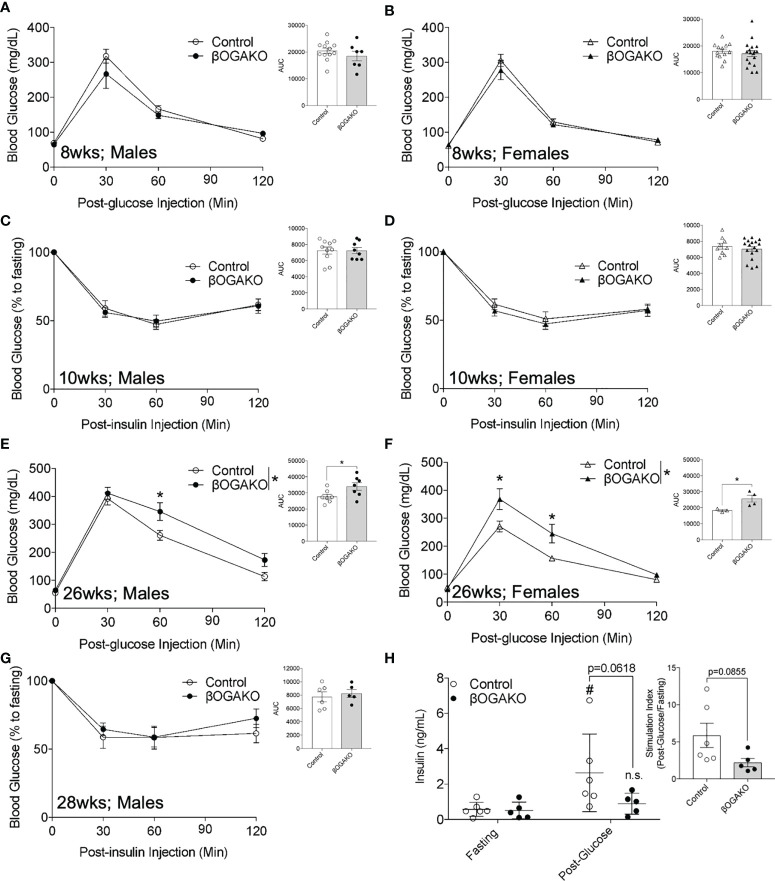
Loss of OGA in β-Cells Leads to Glucose Intolerance in Age-Dependent Manner in normal chow diet. *In vivo* glucose tolerance (2 g/kg glucose, i.p.) test of 8-wks (**A**, Male, n=7-12; **B**, Female, n=13-17) and insulin sensitivity (0.75 U/kg insulin, i.p.) of 10-wks (**C**, Male, n=8-10; **D**, Female, n=9-15). Glucose tolerance test of 26-wks (**E**, Male, n=7; **F**, Female, n=3-4) old mice and insulin of 28-wks old male (**G**, n=5-6) mice. *In vivo* glucose stimulated insulin secretion of male (**H**, n=5-6) with calculated stimulation index. Area under curve (AUC) of the blood glucose curves are presented for each figure. Statistical analyses were conducted using two-way ANOVA and unpaired, 2-way student t-test with significance *p < 0.05, #p<0.05 relative to fasting control. ns, non-statistical significance.

To test the requirement of β-cell OGA in regulating whole body glucose homeostasis, we monitored the following parameters over time: body weight, blood glucose, and glucose and insulin tolerance *in vivo*. βOGAKO exhibited no differences in body weights and non-fasted blood glucose in both males (**Supplemental Figures 1C, E** **)** and females (**Supplemental Figures 1D, F** **)** compared to control mice. At 8-10-wks of age, male and female βOGAKO mice exhibited normal glucose and insulin tolerance when compared to control mice (**Figures 2A-D**). However, at 26wks of age, male and female βOGAKO mice exhibited glucose intolerance (**Figures 2E, F** **)**, without gross alterations to insulin tolerance (**Figure 2G**). Consistently, in response to bolus of glucose treatment, βOGAKO mice failed to significantly increase insulin secretion, compared to control mice (**Figure 2H**), suggesting insulin secretion deficit as the driver of glucose intolerance at this age. Altogether, these data suggest that OGA deletion significantly alters basal β-cell function in both male and female mice under normal chow diet in an age-dependent manner.

### Normal β-cell mass but insulin secretion deficits in male and female βOGAKO mice

**Figure 3:**
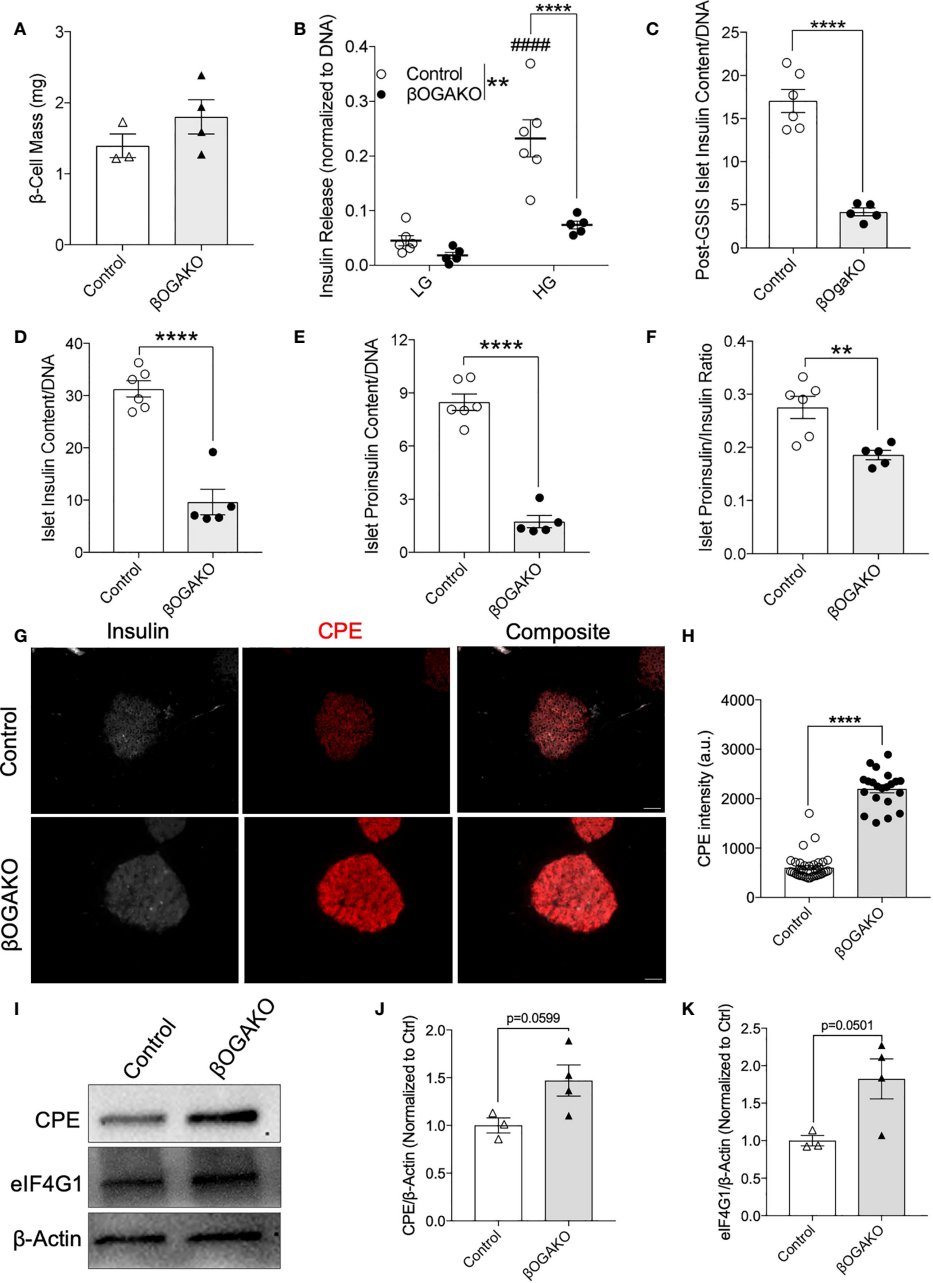
Impaired insulin secretion and processing in β-cell OGA deficient islets. β-cell mass of Control and βOGAKO female mice at 30-wks of age (**A**, n=3-4). *In Vitro* glucose stimulated insulin secretion assay with isolated primary islets from control and βOGAKO mice at 30-wks of age **(B)** with post-assay insulin content normalized to DNA **(C)** (n=5-6). Primary islet insulin **(D)** and proinsulin€) content normalized to DNA with proinsulin to insulin content ratio **(F)** (5-6). Immunofluorescence image of pancreatic islets showing CPE (red) and insulin (white) from Control and βOGAKO pancreas **(G)** and intensity quantification of CPE in insulin positive cells **(H)**. Islet analysis from n=2-3 animals. 40x magnification. Scale bar = 50 μm. Representative western blot **(I)** and quantification of CPE **(J)** and eIF4G1 **(K)**, normalized to beta-Actin (n=3-4). Statistical analyses were conducted using two-way ANOVA and unpaired, 2-way student t-test with significance *p<0.05, ** p<0.001, ****p<0.0001, ^####^pp<0.0001 relative to control fasting.

To further investigate the observed insulin secretion response *in vivo*, we assessed β-cell mass and function *ex vivo*. βOGAKO mice showed no alterations to pancreas mass, β-cell ratio, or β-cell mass (**Figure 3A** **;** **Supplemental Figures 1G, H** **)**. Subsequently, we assessed the secretory function. βOGAKO islets secreted less insulin in response to glucose stimuli than the control islets (**Figure 3B**), and this was in part due to loss in insulin content in these islets (**Figure 3C**). With further analysis, we observed that proinsulin content and proinsulin to insulin ratio was decreased in βOGAKO islets, suggesting reduced proinsulin synthesis or enhanced processing in these islets (**Figures 3D-F**). In our previous study using the βOGTKO model, we showed that the expression of carboxypeptidase E (CPE), a proinsulin cleaving enzyme, was reduced and regulated indirectly by the O-GlcNAcylation of a translational factor, eIF4G1 (17). Consistent with the βOGTKO model, we show that increased O-GlcNAcylation in βOGAKO islets led to increased CPE protein levels (**Figures 3G-J**), concomitantly with increased eIF4G1 protein expression (**Figures 3I, K** **)**. Alterations to CPE and eIF4G1 protein expressions were observed without any significant changes to the transcript levels of CPE and eIF4G1 (**Supplemental Figures 1I, J** **)**, highlighting the O-GlcNAcylation impact of these targets at the post-transcriptional level. Altogether, our data suggests that OGA deletion impacts insulin biosynthesis, processing, and secretory pathways, but not β-cell mass.

### Male and female βOGAKO mice develop glucose intolerance in high fat diet feeding

**Figure 4:**
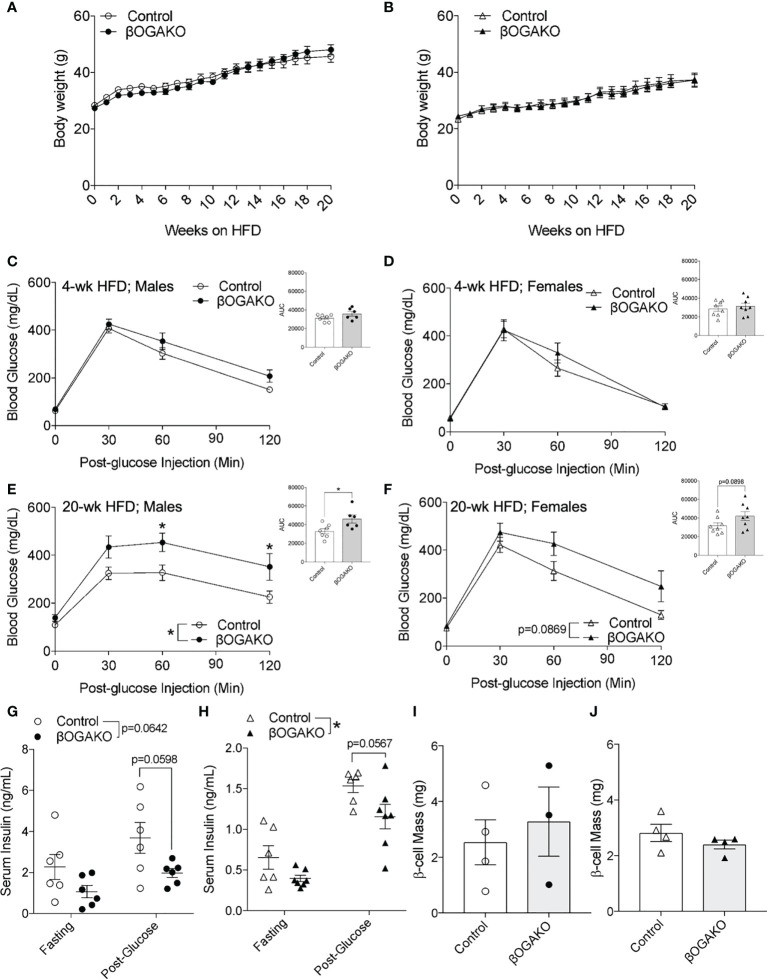
Perturbed glucose homeostasis in βOGAKO mice under high fat diet feeding. Body weight over 20-wks of high-fat-diet (HFD) feeding between control and βOGAKO male (**A**, n=6-7) and female (**B**, n=8) mice. *In vivo* glucose tolerance (2 g/kg glucose, i.p.) test on 4-wks (**C**; Male, **D**; Female) and 20-wks (**E**; Male, **F**; Female) post-HFD (n=6-7 for males and n=8 for females). *In vivo* glucose stimulated insulin secretion of male (**G**, n=6) and female mice 30-wk post-HFD (**H**, n=6-7). β-cell mass, assessed at 32-wk post-HFD in male (**I**, n=3-4) and female mice (**J**, n=4). Statistical analyses were conducted using two-way ANOVA and unpaired, 2-way student t-test with significance *p < 0.05.

To test their propensity to metabolic stress, male and female βOGAKO and littermate control mice were placed on a high-fat diet (HFD; 60% kcal) feed. We detected comparable body weight gain between the genotypes in both male and female mice (**Figures 4A, B** **)**. Echo-MRI imaging performed at 30-wks post-HFD feeding corroborates this data, showing no alterations in fat or lean mass in either male or female βOGAKO mice (**Supplemental Figures 2A-D**). In early HFD feeding (4-wks), no alterations in glucose tolerance were observed in either male or female βOGAKO mice (**Figures 4C, D** **)**. In contrast to normal glucose handling in early HFD, male βOGAKO mice developed significant glucose intolerance with female mice showing strong trends towards intolerance at 20-wks post-HFD feeding (**Figures 4E, F** **)**. The development of glucose intolerance in βOGAKO mice became more severe by the effect of HFD feeding, when compared to normal chow diet (**Supplemental Figures 2H**). No differences were observed in insulin tolerance between control and βOGAKO mice fed a HFD regardless of sex (**Supplemental Figures 2E, F** **)**. Subsequently, we assessed β-cell function *in vivo*. In early HFD feeding (6-wks), we found no significant differences in insulin secretion between control and βOGAKO mice (**Supplemental Figure 2G**). The deficit in insulin secretion in both male and female βOGAKO mice became apparent in longer HFD feeding (30-wks) (**Figures 4G, H** **)**. However, there were no alterations to β-cell mass of βOGAKO mice post-32wk HFD feeding (**Figures 4I, J** **)**, suggesting a defect in insulin secretory capacity. Altogether, these data suggest that hyper-O-GlcNAcylation in β-cells exacerbates the dysfunction in glucose homeostasis and insulin secretion under a diet stressor.

### βOGAKO and littermate control mice display similar response to streptozocin stress

**Figure 5:**
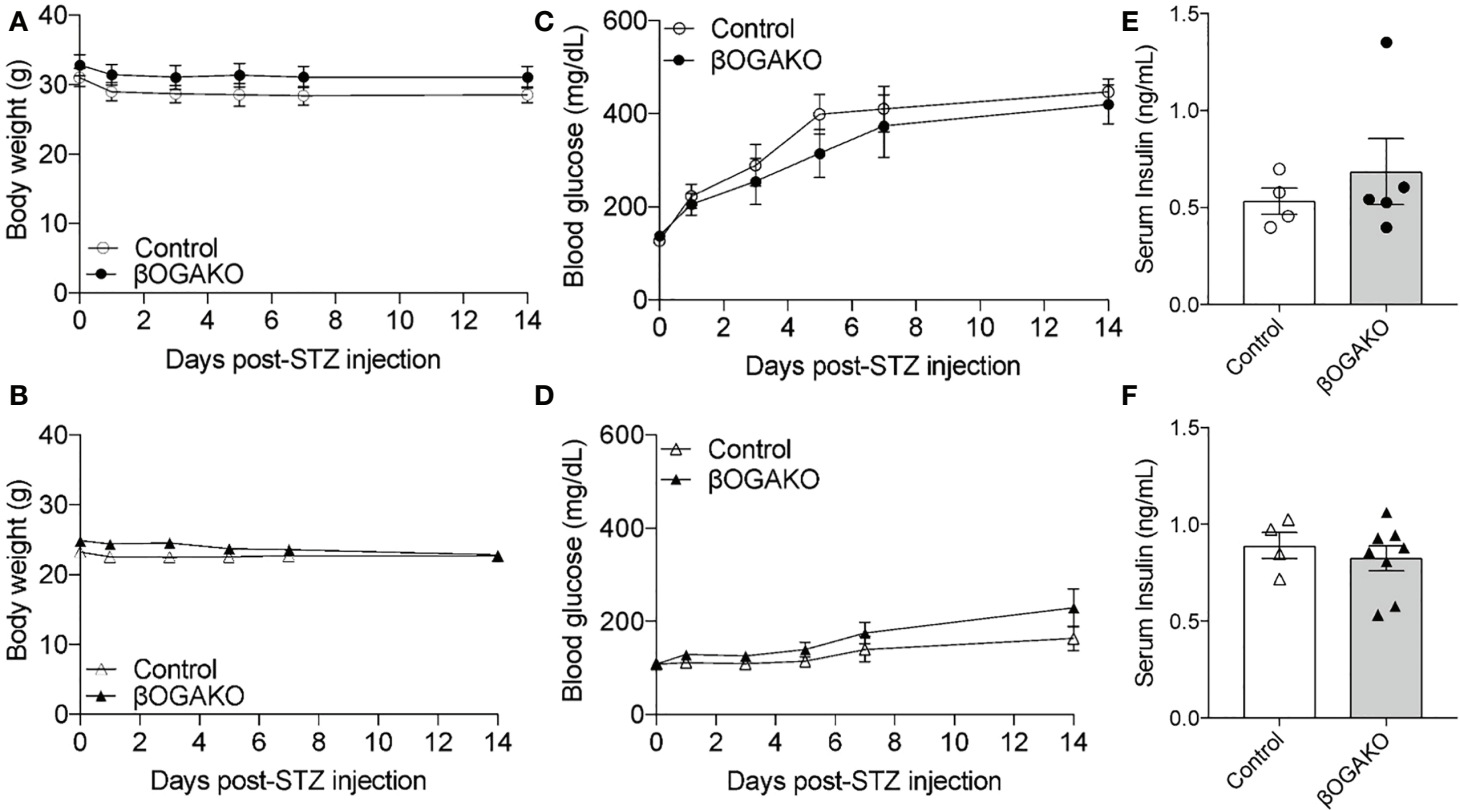
Normal response to diabetogenic STZ treatment in βOGAKO mice. Body weight (**A**, Male; **B**, Female) and blood glucose (**C**, Male; **D**, Female) tracked over 2-wks post-STZ injection (n=5-6 for Males, n=5-8 for Females). Serum insulin (**E**, Male; **F**, Female) at 2-wks post-STZ injection (n=4-5 for Males, n=4-8 for Females). Statistical analyses were conducted using two-way ANOVA and unpaired, 2-way student t-test with significance *p < 0.05.

Streptozocin (STZ) is a common diabetogenic drug to induce hyperglycemia through death of pancreatic β-cells. One mechanism of action by STZ is the inhibition of OGA (17). We hypothesized that deletion of OGA may predispose these mice to diabetes by STZ treatment. To test this hypothesis, 8-10 wk old mice were treated with low-dose STZ injection for 5 days. Body weight, blood glucose and serum insulin were assessed for 2-wks post injection. In both sex, body weight remained normal across control and βOGAKO mice (**Figures 5A, B** **)**. In male mice, control and βOGAKO mice developed hyperglycemia at an equal rate as the controls (**Figure 5C**). As expected, with low dose STZ treatment, the female mice maintained relatively normal blood glucose with no differences between the genotypes (**Figure 5D**). Consistent with the blood glucose levels, the serum insulin level between control and βOGAKO mice remained similar (**Figures 5E, F** **)**. These data suggest that OGA deletion did not alter the mice’s response to low dose STZ stressor relative to littermate controls.

## Discussion

Previous studies have established that O-GlcNAcylation is required for proper β-cell function and metabolic regulation (17). However, it is not known whether dysregulated O-GlcNAc cycling through perturbation of OGA leads to alterations to insulin synthesis and secretion in pancreatic β-cells. To assess the relevance of OGA in β-cell function, we generated β-cell specific OGA knock-out mice. Hyper-O-GlcNAcylation appeared to have no impact on glucose tolerance until 28wks of age, where the animals develop glucose intolerance in part due to reduced glucose stimulated insulin secretion. This deficit in circulating insulin was in part due to dysfunction in β-cell function, but not in mass. The reduction in insulin secretion was associated with decreased total insulin content. To test the loss of OGA against metabolic challenge, we placed these mice in HFD for 25-wks. Body weight did not change for the duration of the metabolic challenge, but glucose intolerance worsened, with insulin secretion deficit, compared to mice fed normal chow diet. In response to the diabetogenic stressor, STZ, we detected no difference in susceptibility to diabetes in either male or female βOGAKO compared to controls, suggesting that HFD, but not STZ, heightened metabolic dysfunction in these mice.

Diabetes and hyperglycemia are often associated with increased O-GlcNAcylation and subsequent tissue dysfunction (1, 22). For example, increased O-GlcNAc modification in islets from diabetic Goto-Kakizaki rats is associated with loss of insulin secretion (23). Genetic deletion of OGA in tissues (such as liver and kidney using MMTV-Cre; OGA flox/+) led to sex-dependent differential metabolic phenotypes in both normal chow and high-fat diet (HFD) feeding (19). While this model is not a conditional deletion of OGA in pancreatic β-cells (24), a sexual-dimorphism in glucose homeostasis dysfunction under metabolic stress was apparent (increased body weight and increased insulin secretion in normal chow diet, and glucose intolerance in HFD despite increased insulin secretion in female, whereas, improved glucose tolerance without changes in insulin secretion in male fed HFD). Interestingly, in our model where OGA is genetically ablated specifically in β-cells, we detected similar metabolic phenotypes in both male and female βOGAKO mice in normal chow and HFD. In males, βOGAKO mice exhibited impaired glucose tolerance with deficit in insulin secretion in either chow diets, unlike normal and impaired glucose tolerance observed in the whole-body deletion model in normal chow or HFD, respectively. In females, βOGAKO consistently showed impairment in insulin secretion; yet whole-body deletion of OGA has led to increased insulin secretion in response to glucose. These data suggest that glucose tolerance and insulin secretion phenotypes in the whole body OGA deletion arises from non-β-cell OGA deficits. Consistent with this idea of differential effects of tissue specific perturbation of O-GlcNAc cycling, specific deletion of OGA in the brain leads to higher insulin in circulation (25). These data highlight the complexity of the glucose homeostasis and maintenance of circulating insulin *in vivo*.

In pancreatic β-cells, deletion of OGT, a model of hypo-O-GlcNacylation, led to the development of diabetes in mice with deficits in both β-cell mass and function (10). In the current model, deletion of OGA in β-cells led to glucose intolerance and insulin secretion deficits in older mice, though animals did not develop hyperglycemia or overt diabetes. These data suggest that perturbed cycling of O-GlcNAcylation on target proteins, whether it be too much or too little, is detrimental for β-cell function. In response to high glucose stimulation, βOGAKO mice failed to respond with adequate insulin secretion. We determined that this defect is in part due to reduced insulin content. In βOGAKO islets, pro-insulin levels and pro-insulin to insulin ratio was reduced, and this is associated with increased expression of prohormone processing enzyme carboxypeptidase E (CPE), suggesting increased pro-insulin processing with possible reduction in pro-insulin transcription or translation. In our previous work in βOGTKO islets, a model of hypo-O-GlcNAcylation, we found that reduced insulin content occurred in part due to disruption in insulin transcription (decrease *Ins1/2* mRNA and Pdx1 protein) as well as proinsulin processing, through dysregulation of CPE *via* O-GlcNAcylation of eIF4G1 (26). These data suggest that insulin processing in part through CPE and eIF4G1 is dependent on the presence of O-GlcNAcylation, where absence leads to reduced processing, while an increase in this PTM leads to enhanced proinsulin processing. However, given that insulin content is reduced in both βOGTKO and βOGAKO islets, overall insulin biosynthesis may be more dependent on O-GlcNAc cycling where either too little or too much O-GlcNAcylation inhibits this process and subsequent insulin secretion.

In addition to a HFD metabolic stressor, we utilized a diabetogenic stressor, streptozocin (STZ) to study the susceptibility of β-cell death under the condition of perturbed O-GlcNAc cycling. STZ is a GlcNAc analog that is selectively toxic to β-cells and one mechanism of action is the inhibition of OGA (27). STZ treatment increase O-GlcNAcylation in β-cells and is associated with β-cell death as transgenic mice with blunted glucosamine synthesis are resistant to STZ effects (28). In our previous study, we showed that transgenic overexpression of OGT in β-cells, a model of hyper-O-GlcNAcylation, led to protection against STZ in female mice (29). However, in the current of model of increasing O-GlcNAcylation *via* deletion of OGA in β-cells, mice exhibited typical diabetogenic effects from STZ, suggesting that STZ can induce β-cell dysfunction independent of its effect on OGA. Other molecular mechanisms of STZ-induced β-cell death include methylation of DNA, nitric oxide production and oxidative stress (30). Also, it is possible that persistent O-GlcNAcylation of a target proteins require regulated cycling to impact their response to stress (in the case of OGT overexpression), rather than completely blunted PTM cycling (as in βOGAKO mice), highlighting the importance of enzyme kinetics in cellular response and function.

In summary, we demonstrate that ablation of OGA in β-cells causes a defect in insulin secretion but not β-cell mass. Mice lacking OGA in their β-cells develop glucose intolerance with age but not overt diabetes. These mild phenotypes are distinct to that of mice lacking OGT in their β-cells, where they develop severe hyperglycemia and over diabetes in early adulthood in part by decreased β-cell mass and insulin secretion failure. These studies highlight importance of O-GlcNAcylation cycling but also the distinct non-enzymatic actions of OGT and OGA in these cells.

## Experimental procedures

### Animal models and *in vivo* mouse procedures

The following mice were used as breeders for the study: OGA flox/flox (provided by Dr. John Hanover (NIH)), and mice harboring one allele of Cre-recombinase under the rat insulin 2 promoter [Rip-Cre; provided by Dr. Pedro Herrera (University of Geneva)], CAG-GFP Cre-recombinase reporter (Jackson Laboratories). All mice were group housed on a 14:10 light-dark cycle. High-fat diet (HFD; 60% Kcal of fat, D12492) was purchased from Research Diets, Inc. Glucose and insulin tolerance tests and *in vivo* glucose stimulated insulin secretion assays were performed as previously described (10), with littermate (OGA flox/flox, referred to as Control) mice. Low dose streptozocin (50 mg STZ/kg bw) was injected for 5-consecutive days and the mice were studied for 2-wks to assess metabolic parameters. All procedures were performed in accordance with the University of Minnesota Animal Studies Committee (IACUC #1806A36072).

### Islet isolation and insulin secretion assay

We have previously described our islet isolation and insulin secretion assay technique (10). In brief, islets were isolated following ductal perfusion of collagenase and handpicked into RPMI media (10% FBS, 5 mM glucose) for overnight culture before experimental use. For the secretion assay, 3x10 islet aliquots per mouse were sequentially incubated in Krebs buffer at low glucose (2 mM, LG, 30 min), HG (high glucose, 16.7 mM, 30 min) and 30 mM KCl (15 min). Islet were collected into RIPA buffer (CST) with protease inhibitor cocktail (CST). Insulin secretion is presented as % of post-secretion islet insulin content. Total islet insulin content was calculated as the sum of secreted insulin + insulin content of the remaining islets, normalized to DNA.

### Insulin ELISA

Insulin and proinsulin levels from random-fed serum, lysed isolated islets and islet secretion solutions were measured using Mouse Ultrasensitive Insulin ELISA kit (Alpco; 80-INSMSU-E01) per kit instruction. Content data was normalized to DNA, as determined by Quant-iT Pico Green dsDNA Assay (Molecular Probes).

### Western blot

Primary pancreatic islets were lysed by sonication in 1x RIPA buffer, supplemented with protease and phosphatase inhibitor cocktails (CST). Upon BCA protein analysis, protein lysates were resolved by SDS-PAGE, followed by transfer to PVDF membrane. Membrane was blocked with 5% non-fat dry milk and probed with following antibodies: RL2 (Abcam; ab2739), OGA (Sigma; SAB4200311), OGT (CST; 24083), CPE (BDScience; 610759), eIF4G1 (Proteintech; 15704-1-AP), Actin (CST; 4967).

### qPCR analysis

RNA was isolated from pancreatic islets, using RNeasy plus micro kit, following manufacturer’s protocol. For qPCR, cDNA was synthesized from islet RNA, using high-capacity cDNA reverse-transcription kit (Applied Biosystems). Relative gene expression was assessed with Sybr Green (Applied Biosystems) on QuantStudio 6 Flex Real-Time PCR systems and calculated with ΔΔ cycle threshold (ΔΔCT) normalized to loading control. Primer sequences are: OGA Forward (TACCTGGGAGAGCCAGAAAC), OGA Rev (TGGATAACAGAAAGTGCCACA), CPE Forward (GCTCAGGTAATTGAAGTCTT), CPE Rev (TACTGCTCACGAATACAGTT), OGT Forward (ACTGTGTTCGCAGTGACCTG), OGT Rev (TCAAATAACATGCCTTGGCT), eIF4G1 Forward (TGGGAGGCTGATTCTCTACC), eIF4G1 Rev (GGAGACCTTCTAGATGCCA), Actin Forward (GCCCTGAGGCTCTTTTCCAG), and Actin Rev (TGCCACAGGATTCCATACCC).

### Immunofluorescence & β-cell mass analysis

5 µm sections were generated at intervals of 200 µm from 5 different regions of each formalin-fixed and paraffin-embedded adult pancreas. Tissue sections were then selected from each region for staining. Following deparaffinization, antigens were retrieved by microwaving tissues in 0.01 M sodium citrate/citric acid for 12 minutes at 95C. Sections were then permeabilized with 0.01% Triton and blocked with Roche blocking buffer. Tissues were incubated overnight at 4°C in primary antibodies against guinea pig insulin, mouse glucagon, and DAPI. Tissues were then washed with PBS-0.01% Tween and incubated with FITC, Cy3, and AMCA-conjugated secondary antibodies for 2 hours at 37°C. DAPI staining was also performed according to instructions provided by the manufacturer. β-cell mass was performed as previously described (10). For intensity analysis, all slides were imaged at the same time under equal channel exposure conditions. Using ImageJ (v1.53q), each image was thresholded such that only the islets were selected, and absolute average intensity was measured for each islet.

### Statistical analysis

Data are presented as mean ± SEM and were analyzed using 2-tailed unpaired Student t-test. Multiple outcome data were assessed using repeated measures 2-way ANOVA. Statistical analyses were performed in GraphPad Prism version7 with a significance threshold of p<0.05.

## Data availability statement

The original contributions presented in the study are included in the article/**Supplementary Material**.

## Ethics statement

The animal study was reviewed and approved by IACUC at UMN.

## Author contributions

Developed the study, SJ and SP. Designed experiments, generated and analyzed data, assisted with manuscript preparation, and approved the final version, SJ, SP, NA, AW, AE, JH, and EA. Interpreted the data and wrote and edited the manuscript, SJ and EA. Conceived the study and in charge of overall direction of this work, EA. All authors contributed to the article and approved the submitted version.

## Funding

This work was supported by National Institutes of Health Grant NIDDK (R21DK112144 and R01DK115720 to EUA; F31DK131860 to SJ, T32 DK007203 for SP; and T32GM140936 for AW). This work was also supported by the Department of Integrative Biology and Physiology Accelerator Program.

## Acknowledgments

We acknowledge Dr. Amber Lockridge, Ms. Grace Chung, and Mr. Nicholas Esch for technical support. We thank Dr. Thomas Pengo for his assistance in Fiji and the University of Minnesota Imaging Center for technical support. We thank Dr. Pilar Ariza Guzman for MRI in IBP phenotyping core at University of Minnesota. The tissue processing and embedding was performed at the laboratory of Dr. Jop van Berlo.

## Conflict of interest

The authors declare that the research was conducted in the absence of any commercial or financial relationships that could be construed as a potential conflict of interest.

## Publisher’s note

All claims expressed in this article are solely those of the authors and do not necessarily represent those of their affiliated organizations, or those of the publisher, the editors and the reviewers. Any product that may be evaluated in this article, or claim that may be made by its manufacturer, is not guaranteed or endorsed by the publisher.

## Supplementary material

Supplementary Figure 1 Normal Chow Diet Phenotype. OGT mRNA level from pancreatic islets of control and βOGAKO mice **(A)**, normalized to beta-Actin mRNA (n=3-4). Representative western blot and quantification of OGT **(B)**, normalized to beta-Actin (n=3-4). Body weight from 5- to 15-wks of age in male **(C)** and female **(D)** mice under normal chow diet (n=5-6 for males, n=8-11 for females). Non-fasted blood glucose at 15-16 wks of age in male **(E)** and female **(F)** mice (n=6-7 for males, n=4 females). Pancreas mass **(G)** and β-cell area to pancreas area ratio **(H)** from control and βOGAKO mice (n=3-4). CPE **(I)** and eIF4G1 **(J)** mRNA level from pancreatic islets of control and βOGAKO mice, normalized to beta-Actin mRNA (n=3-4). Statistical analyses were conducted using two-way ANOVA and unpaired, 2-way student t-test with significance *p<0.05

Supplementary Figure 2 High Fat Diet Phenotype. Echo-MRI analysis of fat mass and lean mass for male **(A, B)** and female **(C, D)** mice (n=6-7 for males and n=8 for females). Insulin sensitivity (0.75 U/kg insulin, i.p.) on 24-wks post-HFD for male **(E)** and female **(F)** mice. (n=6-7 for males and n=8 for females). *In vivo* GSIS of mice on 6-wks HFD (**G**) (n=4-7). IPGTT AUC from and comparing normal chow and HFD conditions **(H)**.
