# Supplementary figures and images for: Pancreatic β-cell hyper-O-GlcNAcylation leads to impaired glucose homeostasis in vivo
Source: Front Endocrinol (Lausanne). 2022 Oct 26;13:1040014. doi: 10.3389/fendo.2022.1040014 (PMC9644030; doi:10.3389/fendo.2022.1040014)

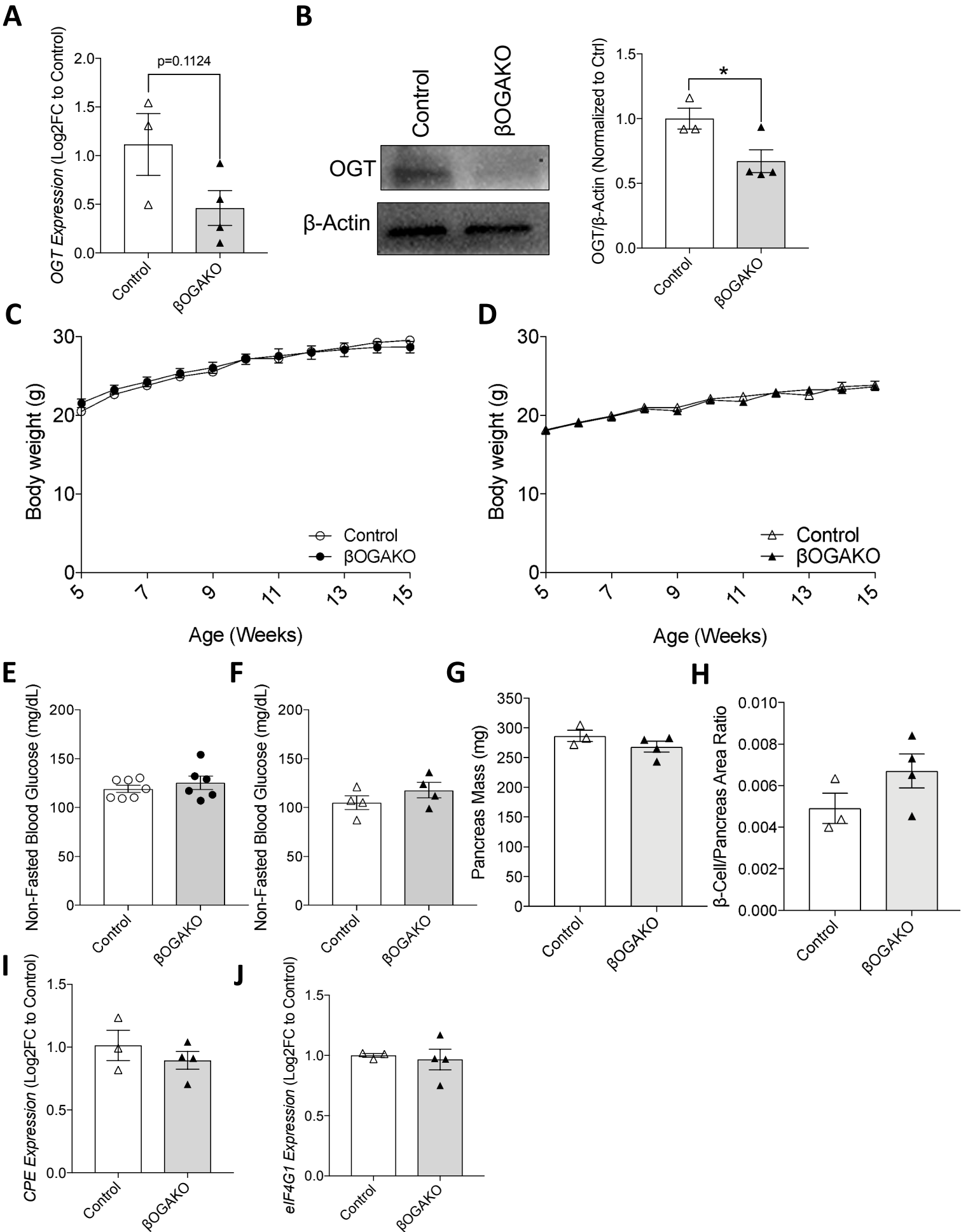

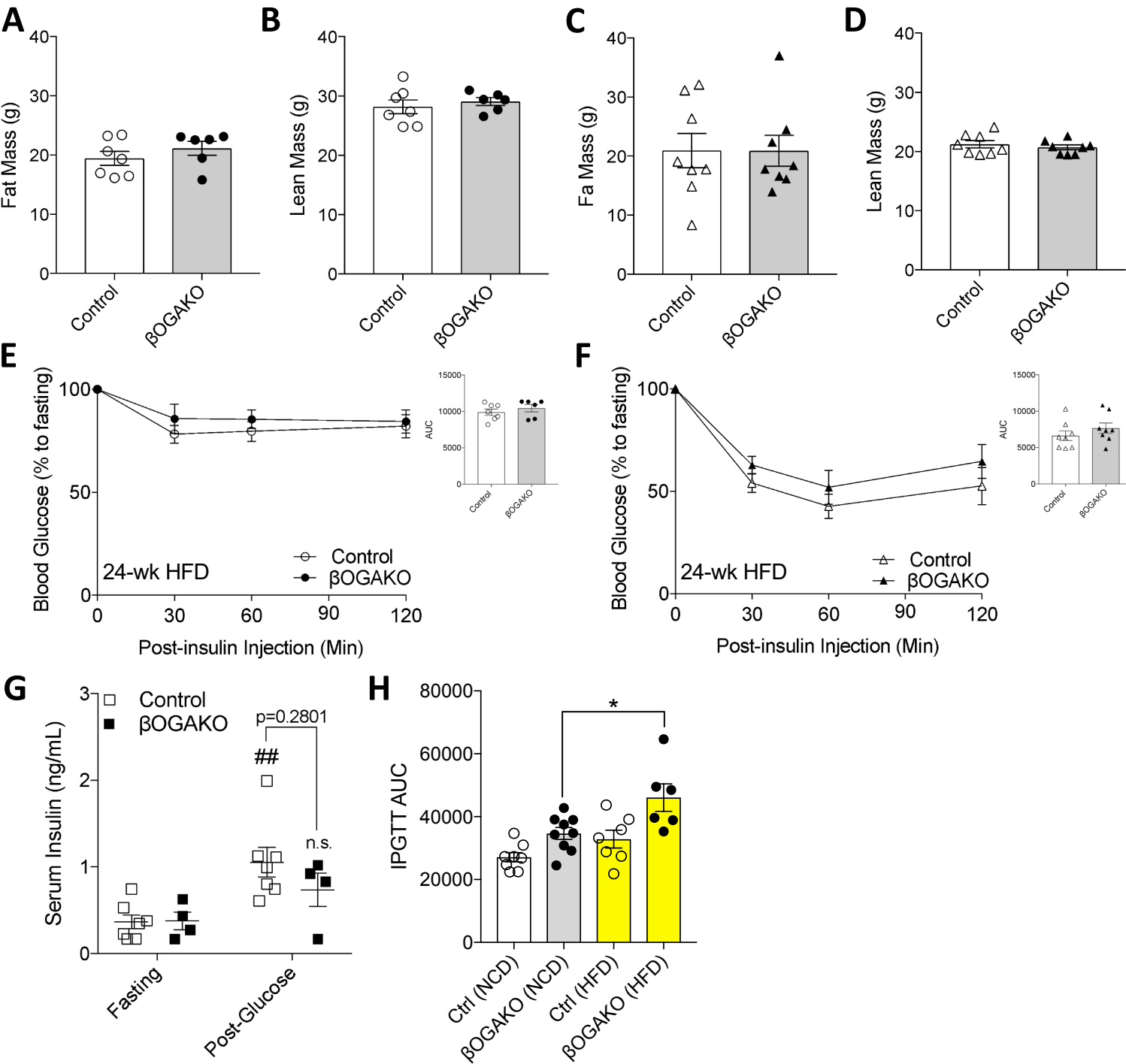

Supplement: Supplementary Figure 1 — Normal Chow Diet Phenotype. OGT mRNA level from pancreatic islets of control and βOGAKO mice (A), normalized to beta-Actin mRNA (n=3-4). Representative western blot and quantification of OGT (B), normalized to beta-Actin (n=3-4). Body weight from 5- to 15-wks of age in male (C) and female (D) mice under normal chow diet (n=5-6 for males, n=8-11 for females). Non-fasted blood glucose at 15-16 wks of age in male (E) and female (F) mice (n=6-7 for males, n=4 females). Pancreas mass (G) and β-cell area to pancreas area ratio (H) from control and βOGAKO mice (n=3-4). CPE (I) and eIF4G1 (J) mRNA level from pancreatic islets of control and βOGAKO mice, normalized to beta-Actin mRNA (n=3-4). Statistical analyses were conducted using two-way ANOVA and unpaired, 2-way student t-test with significance *p<0.05 [file Image_1.pdf]
